# Average demands and most demanding passages of national-level female soccer matches: do small- and large-sided games replicate match demands?

**DOI:** 10.3389/fspor.2023.1236112

**Published:** 2023-10-11

**Authors:** E. H. J. Savolainen, T. Vänttinen, J. Peltonen, J. K. Ihalainen, S. Walker

**Affiliations:** ^1^ Faculty of Sport and Health Sciences, University of Jyväskylä, Jyväskylä, Finland; ^2^ Finnish Institute of High Performance Sport KIHU, Jyväskylä, Finland; ^3^ Polar Electro Oy, Kempele, Finland; ^4^ NeuroMuscular Research Center, University of Jyväskylä, Jyväskylä, Finland

**Keywords:** football, women, external load, internal load, various-sided games, tactical periodization

## Abstract

**Introduction:**

This study aims to (1) determine the average and most demanding passage (MDP) load of national-level female soccer matches and (2) evaluate the relationship between average and MDP load during small-sided games (SSGs), large-sided games (LSGs), and matches.

**Methods:**

A total of 37 national-level female soccer players from a single club senior team and the U18 team participated. The average and 1-, 3-, and 5-min MDP external (total, high-speed, and very-high-speed running distances, acceleration and deceleration distances, average metabolic power, and high-metabolic load distance) and internal loads (average heart rate, rate of perceived exertion) of the 29 league matches, ten 4 vs. 4 + goalkeepers SSGs, and six 8 vs. 8 + goalkeepers LSGs were analyzed by the Polar Team Pro player tracking system.

**Results:**

In matches, the external load variables during 1-, 3-, and 5-min MDPs were 167%–1,165%, 135%–504%, and 126%–359%, of match average values, respectively. In LSGs, all external load variables reached higher average values compared with those during matches, except for the very-high-speed running distance; however, no variable reached higher values of 1-min MDP compared with those during the matches. In SSGs, the average acceleration and deceleration distances were higher compared with those during the matches.

**Discussion:**

The findings from the present study suggest that LSGs and SSGs can be used to overload the average values of the selected external load variables compared with those during the matches; however, other training options must be explored to overload 1-min match MDPs.

## 1. Introduction

Tactical periodization is a holistic approach to training, where its training ideology does not separate the physiological, tactical, technical, and psychological elements of soccer (1). This approach has become popular in planning and periodizing soccer training (2). From a fitness perspective, the key principle of tactical periodization is to overload three main physical capacities (strength, endurance, and speed), relative to match demands, in a soccer-specific manner within the week (1). Thus, soccer coaches and practitioners must be aware of the match demands and loads of various-sided games that they play as a part of weekly training to sufficiently plan for intensive training to overload and ultimately improve the targeted physical qualities of the players. The majority of previous studies on female soccer players have defined match demands as total (3–5) (e.g., running distance during the match) or time-normalized (6) (i.e., total values divided by the time of play in minutes) demands. Average time-normalized demands heavily underestimate the highest intensity that players perform during the match, at least in elite players, due to the intermittent nature of the soccer match play (7). Thus, an increasing number of studies have defined the most demanding passage (MDP) of a match play (7, 8).

A recent review by Harkness-Armstrong et al. (9) showed that MDPs (or peak demands) of female soccer matches have been typically quantified from pre-determined segments (e.g., 5 min). A few studies have used the rolling average (RA) method in female soccer analysis to quantify match MDPs (9). This is problematic because a segmental approach can underestimate match MDPs by up to 25% compared with the RA method, as observed in male soccer players (10). For example, a 5-min match MDP defined by the segmental approach is reported to be more than twofold for high-speed running distance, threefold for the number of accelerations and decelerations, and three- to sixfold for sprint distance, depending on the playing position, compared with the match average in elite female players (7). However, the difference between the match average and the “real” MDP defined by the RA method is even higher. Although there are methodological differences in defining MDPs, there is convincing evidence that average values underestimate the match demands of the players. In addition, training stimuli to develop the capabilities of the players to perform during the most intensive periods of the match could be insufficient if only average values are used for training prescription and if MDPs are not taken into account. However, most studies to date have focused on senior elite- or top-tier domestic female players; thus, more information is needed from different levels and age groups (9).

Small-sided games (SSGs), medium-sided games (MSGs), and large-sided games (LSGs) are effective training methods that are extensively used in soccer to concurrently simulate and improve the physical, technical, and tactical aspects of the game (11). Several studies have compared SSG average and MDP load to the matches of elite male players (12–16); however, there is a lack of evidence in female players (17). In one study, Gabbett and Mulvey (18) found that 3 vs. 3 and 5 vs. 5 SSGs simulated the average movement patterns of the competition overall, but the stimulus of the repeated sprints for the international competition was insufficient. One major barrier to evidence-based practice is that a comparison between the match load of the female players and LSGs or an association between the MDPs of matches and various-sided games is not documented. Thus, there is no evidence to inform coaching practice on how to utilize SSGs or LSGs to target specific aspects of female match play fitness.

Due to limited knowledge of match MDP demands of female soccer players and how SSGs and LSGs prepare female players in relation to average and MDP match loads, this study aimed (1) to determine the average and MDPs of national-level female soccer matches and (2) to evaluate the relationship between average and MDPs during SSGs, LSGs, and matches. We hypothesized that (1) match average values also underestimate the highest intensity (MDPs) of match play in national-level players (7) and (2) SSGs and LSGs can replicate or overload the selected variables relative to match load, as previously observed in male players (14). Increasing knowledge of these aspects will allow evidence-informed decisions on how to utilize SSGs and LSGs to better prepare female players for future matches.

## 2. Methods

### 2.1. Participants

A total of 37 national-level [see (19) for definition], amateur or semiprofessional, outfield female soccer players from a single club in Finland participated in this study. The players were from two teams: the senior team (*n* = 17, seven defenders, six midfielders, and four attackers, age 21.2 ± 2.6 years, typically five to six training sessions per week during the match season), who played in the highest national league, and the U18 team (*n* = 20, seven defenders, eight midfielders, and five attackers, age 16.9 ± 0.7 years, typically four to six training sessions per week during the match season), who played in the highest U18 national league. All participants, and parents of younger participants aged <18 years, provided written informed consent prior to the study. The participants were informed, both verbally and in written form, of the possible risks and discomforts associated with the study procedures, and they had the opportunity to discuss the study with the researchers. The study was approved by the Ethics Committee of the Health Care District of Central Finland (identification number: 5U/2019, 24 April 2020) and conducted according to the Declaration of Helsinki (2013), except for database registration.

### 2.2. Study design

In this observational study, data such as global positioning system (GPS) and heart rate (HR) were collected from all official league matches (during the 2020 competitive season) and from standardized SSGs (4 vs. 4 + GKs) and LSGs (8 vs. 8 + GKs), which were both played twice during the training sessions. The COVID-19 pandemic affected the training during preseason and delayed the start of the match season by approximately 2 months; however, this did not directly impact the present research data collection. A comparison of SSG and LSG demands with match demands was performed by time-normalized (e.g., m/min) average and MDP values. MDPs were defined by the highest value in the RA method of 1-, 3-, or 5-min time intervals.

### 2.3. Methodology

All league matches from the 2020 season were analyzed. The senior team played 18 matches (five wins, one draw, and 12 losses), and the U18 team played nine matches (six wins, three draws, and two losses). The only difference in rules between competition levels was in the maximum number of substitutions allowed per match, five in the senior team and seven in the U18 team. Match data from the entire match season were used to maximize the sample size and because our analysis showed no statistically significant decrease or increase in match demands during the season. Match data of the players were included in the analyses if the player played more than 75 min in the match (8). In total, a period of 75 min was selected because match demands are highest at the beginning of the match and then begin to decrease toward the end of the match (5) and to minimize the loss of observation numbers, e.g., due to increased rate of substitutions late in the match. Both teams played most of the matches in a 4-4-2 formation. A total of 249 match observations were included in the analyses (senior team = 167, on average 9.8 ± 4.4 per player, and U18 team = 82, on average 4.2 ± 2.1 per player). The average value from the match observations of each player was calculated and used in the final analyses to represent the match demands of each player.

The objective of SSGs and LSGs was to win every game and score as many goals as possible. Spare balls were kept in the goal area of each team in such a way that the goalkeeper could restart the game quickly when a goal was scored or the ball went out of play. Both SSGs and LSGs were played twice inside a 10-week period during the last 3 months of the match season. In a single session, SSGs and LSGs were played 5 min × 3 min and 3 min × 5 min, respectively. Each game was considered as an individual observation, and one value (the average of all observations) was included in the final analyses, as was performed for the matches. A total of 325 observations from SSGs (senior team = 145, on average 8.5 ± 2.5 per player, and U18 team = 180, 9.0 ± 2.0 per player) and 204 samples from LSGs (senior team = 96, on average 5.7 ± 0.9 per player, and U18 team = 108, 5.4 ± 1.2 per player) were collected. The measurements were performed in the team training environment, and, unfortunately, all players did not participate in the same amount of SSG and LSG sessions, as expected.

The games were played as part of the normal team training sessions following a standardized warm-up of 20 min. The players were assigned to level-balanced teams (based on overall physical, technical, and tactical ability) by the coaching staff. SSGs were played as 4 vs. 4 + GKs on a 32 m × 22 m field. The playing time was 5  min × 3 min with 3-min rest intervals between games. LSGs were played as 8 vs. 8 + GKs on a 75 m × 48 m field. The playing time was 3 m × 5 min with 3-min rest intervals between games. The area per player was 94 m^2^ in SSGs and 225 m^2^ in LSG [the goalkeepers were excluded for calculations of the area coverage as in the study of Riboli et al. (13)]. The teams used a 1-2-1 formation in SSGs and a 2-4-2 formation in LSGs. During SSGs, blood lactate concentration was measured immediately after the first, third, and fifth games and after all three games in LSGs. Lactate samples were analyzed by using Biosen S-line Lab + (EKF Diagnostics, Magdeburg, Germany) according to the manufacturer's instructions. The self-reported rate of perceived exertion (RPE, Borg CR-10) of the players was recorded after every game. The average values of RPE and lactate were used in further analyses.

GPS and HR data were collected by the Polar Team Pro player tracking system (Polar Electro Oy, Kempele, Finland) with GPS sampling at 10 Hz. Good-to-moderate reliability (<5% CV) and validity for total distance, linear running, and team sport simulation circuit were shown in the Polar Team Pro system (20). The time-normalized (e.g., m/min) average and MDP values, defined by the highest value in a RA method (1-, 3-, or 5-min time intervals), were used in the analyses. The relative MDP values from the matches were also calculated by dividing the MDP value of the player by the match average value of the player. Finally, the relative MDP values from SSGs and LSGs were calculated by dividing the SSG or LSG MDP value of the player by the match MDP value of the same duration. MDPs were calculated from the Polar Team Pro raw data, which were exported from Polar online software and analyzed by a customized MATLAB script [version: 9.13.0 (R2022b)] to determine the 1-, 3-, and 5-min MDP values from the selected time windows (match, LSG, or SSG duration). Prior to analyses, player data were excluded in cases where it was missing or partly missing from the match, SSG, or LSG, i.e., in situations where the player tracking system did not record data for the whole match (*n* = 21), SSG (*n* = 1), or LSG (*n* = 1) period. From the match data, two matches (both wins) from the U18 team were excluded from analyses due to hardware-related technical problems (*n* = 17 from the above 21). Further, during data review, error peaks were removed from peak heart rates, e.g., well above 100% of the HRmax (*n* = 12 samples overall) (5).

The following variables were used to represent the external load of matches, SSGs, and LSGs: total distance (m/min), HSRD (13–19 km/h) (m/min), very HSRD (VHSRD, >19 km/h) (m/min) (21), distance covered in acceleration (ACCD, >2 m/s^2^) (m/min) and deceleration (DECD, <−2 m/s^2^) (m/min) (22), average metabolic power (Pmet) (W/kg) (23), and distance covered in high metabolic power [high-metabolic load distance (HMLD), >20 W/kg] (m/min) (24).

Average HR (%/max), RPE (1–10), and blood lactate concentration (mmol/L) (measured in SSGs and LSGs, but not in official matches) were measured to represent the internal load.

### 2.4. Statistical analyses

Statistical analyses were conducted using SPSS Statistics 28 (IBM, Armonk, NY, USA). The results were reported as means ± standard deviation (SD). Data normality was assessed using the Shapiro–Wilk test, and all variables used to analyze the average and peak match, SSG, and LSG demands were normally distributed (the following variables after logarithmic transformation: match 1-, 3-, and 5-min VHSRD MDP; match 1-, 3-, and 5-min ACCD MDP; match 1-, 3-, and 5-min HMLD MDP; SSG average and 3- and 1-min MDPs in VHSRD; and SSG 3-min HSRD MDP). SSG and LSG demands relative to match demands (percentage) remained not normally distributed after log transformation, and non-parametric tests were used to analyze each of those variables. Alpha was defined as below 0.05.

Independent sample *t*-tests were used to investigate the possible differences between the senior team and U18 team players in match average, MDP, and relative MDP values. Independent sample *t*-tests were also used to analyze the possible differences between the SSG and LSG average and MDP values of the senior and U18 teams. For pairwise comparisons, the effect sizes were calculated by using Hedges’ *g* and were classified using the following criteria: 0.2–0.5, small; 0.5–0.8, medium; and >0.8, large.

Repeated measures ANOVA (group × game type) and Bonferroni *post-hoc* tests were used to investigate the possible differences between match, SSG, and LSG average and MDP values. The Mann–Whitney *U*-test was used to investigate the possible differences in SSG and LSG demands relative (percentage) to match load between the senior and U18 teams. Finally, the Wilcoxon signed-rank test was used to examine the possible differences between SSG and LSG demands relative to match load.

The between-match, between-SSG, and between-LSG session coefficient of variation (CV) were calculated to evaluate intersession variability. CVs are presented in Tables 1–3.

**Table 1:** Average and MDPs of match demands of the senior team and U18 team players.

Senior team	Match average		Match 5-min MDP				Match 3-min MDP				Match 1-min MDP			
	Mean + SD (*g*)	CV	Mean + SD (*g*)	% of match-avg (*g*)	*r*	CV	Mean + SD (*g*)	% of match-avg (*g*)	*r*	CV	Mean + SD (*g*)	% of match-avg (*g*)	*r*	CV
TD (m/min)	107.8 ± 7.3* **(1.05)**	5.0 ± 2.7	135.9 ± 9.3* (0.76)	126 ± 4* **(−0.86)**	**0.91**	5.2 ± 1.9	145.3 ± 9.9* **(0.89)**	135 ± 4 (−0.61)	**0** **.** **88**	4.4 ± 1.5	180.0 ± 13.6 (0.67)	167 ± 7* (−0.78)	**0** **.** **80**	6.1 ± 2.1
HSRD (13–19 km/h) (m/min)	21.2 ± 4.8* (0.94)	11.9 ± 4.9	39.1 ± 7.0* **(0.90)**	187 ± 18 (−0.40)	**0.93**	18.8 ± 9.6	47.3 ± 7.5* **(0.93)**	227 ± 24 (−0.46)	**0** **.** **92**	18.9 ± 10.0	82.1 ± 12.7* **(1.09)**	395 ± 49 (−0.22)	**0** **.** **85**	19.0 ± 12.0
VHSRD (>19 km/h) (m/min)	4.4 ± 1.8 (0.63)	25.9 ± 10.8	14.8 ± 5.0* **(0.87)**	352 ± 64 (−0.09)	**0** **.** **95**	31.3 ± 11.3	20.7 ± 6.6* **(0.91)**	498 ± 112 (−0.05)	**0** **.** **95**	30.4 ± 11.3	44.5 ± 12.4* **(0.80)**	1,081 ± 245 (−0.29)	**0** **.** **63**	26.8 ± 11.1
Pmet (W/kg)	7.4 ± 0.5* (1.09)	4.9 ± 2.7	9.4 ± 0.7 (0.71)	127 ± 4* **(−0.97)**	**0** **.** **92**	5.4 ± 2.1	10.1 ± 0.8* **(0.81)**	136 ± 4* **(−0.86)**	**0** **.** **92**	4.4 ± 2.2	12.6 ± 1.1 (0.65)	171 ± 8* (−0.76)	**0** **.** **83**	4.9 ± 2.2
HMLD (>20 W/kg) (m/min)	12.6 ± 2.8* (0.76)	11.6 ± 3.9	24.1 ± 5.0* **(0.92)**	193 ± 19 (0.08)	**0** **.** **87**	15.0 ± 11.8	30.0 ± 6.1* **(0.93)**	241 ± 26 (0.05)	**0** **.** **84**	15.7 ± 12	51.3 ± 9.6* (0.72)	414 ± 51 (−0.09)	**0** **.** **83**	16.3 ± 11.8
ACCD (>2 m/s^2^) (m/min)	2.2 ± 0.6* **(0.83)**	18.0 ± 5.6	4.6 ± 0.9* **(0.99)**	216 ± 30 (0.08)	**0** **.** **87**	24.4 ± 11.4	5.7 ± 1.2* **(0.98)**	266 ± 34 (0.01)	**0** **.** **88**	22.2 ± 12.7	10.3 ± 1.8* **(0.90)**	480 ± 66 (−0.23)	**0** **.** **79**	21.3 ± 12.5
DECD (<−2 m/s^2^) (m/min)	2.0 ± 0.5 (0.62)	13.8 ± 9.5	4.0 ± 0.7* (0.75)	209 ± 22 (0.13)	**0** **.** **91**	18.1 ± 12.3	5.0 ± 1.0* (0.78)	262 ± 34 (0.08)	**0** **.** **85**	17.7 ± 11.3	9.0 ± 1.5* **(0.91)**	473 ± 71 (0.04)	**0** **.** **83**	18.6 ± 11.0
HR (%/max)	85.6 ± 2.4 (0.04)	2.6 ± 1.4	90.6 ± 2.3 (−0.30)	106 ± 1 (−0.5)	**0** **.** **91**	2.4 ± 1.6	91.8 ± 2.1 (−0.17)	107 ± 2 (−0.30)	**0** **.** **85**	2.1 ± 1.5	93.7 ± 2.3 (0.02)	109 ± 3 (−0.02)	**0** **.** **59**	1.6 ± 0.8
RPE (1–10)	8.1 ± 0.7* (0.34)	12.3 ± 4.8												
U18 team	Match average		Match 5-min MDP				Match 3-min MDP				Match 1-min MDP			
	Mean + SD	CV	Mean + SD	% of match-avg	*r*	CV	Mean + SD	% of match-avg	*r*	CV	Mean + SD	% of match-avg	*r*	CV
TD (m/min)	98.9 ± 9.3	6.0 ± 3.3	128.3 ± 10.5	130 ± 5	**0** **.** **91**	4.0 ± 1.6	136.2 ± 10.6	138 ± 6	**0** **.** **90**	3.9 ± 1.8	171.1 ± 12.7	173 ± 9	**0** **.** **83**	5.6 ± 2.9
HSRD (13–19 km/h) (m/min)	16.9 ± 4.3	18.0 ± 18.1	32.6 ± 7.3	195 ± 25	**0** **.** **82**	26.1 ± 21.1	39.9 ± 8.2	241 ± 35	**0** **.** **77**	26.9 ± 20	67.4 ± 14.2	408 ± 68	**0** **.** **72**	30.2 ± 19
VHSRD (>19 km/h) (m/min)	3.3 ± 1.5	37.9 ± 21.2	10.9 ± 4.0	359 ± 92	**0** **.** **97**	40.8 ± 22.8	15.2 ± 5.3	504 ± 133	**0** **.** **92**	42.4 ± 20.0	34.9 ± 11.7	1,165 ± 325	**0** **.** **68**	38.9 ± 20.6
Pmet (W/kg)	6.7 ± 0.7	8.6 ± 7.8	8.9 ± 0.7	133 ± 8	**0** **.** **87**	4.7 ± 2.0	9.5 ± 0.7	142 ± 8	**0** **.** **88**	5.6 ± 2.0	12.0 ± 0.9	180 ± 14	**0** **.** **71**	5.5 ± 2.8
HMLD (>20 W/kg) (m/min)	10.5 ± 2.8	16.7 ± 19.3	19.7 ± 4.5	192 ± 24	**0** **.** **87**	26.6 ± 21.5	24.5 ± 5.6	239 ± 34	**0** **.** **83**	29.9 ± 17.8	43.4 ± 12.1	419 ± 63	**0** **.** **81**	30.0 ± 19.7
ACCD (>2 m/s^2^) (m/min)	1.8 ± 0.5	22.6 ± 20.3	3.7 ± 1.0	214 ± 26	**0** **.** **98**	31.5 ± 26.3	4.6 ± 1.2	266 ± 43	**0** **.** **90**	31.7 ± 25.3	8.5 ± 2.1	502 ± 113	**0** **.** **74**	33.4 ± 26.4
DECD (<−2 m/s^2^) (m/min)	1.6 ± 0.6	22.9 ± 24.5	3.3 ± 1.2	205 ± 41	**0** **.** **81**	29.6 ± 27.5	4.1 ± 1.3	258 ± 52	**0** **.** **84**	30.7 ± 26.4	7.3 ± 2.2	469 ± 126	**0** **.** **73**	30.7 ± 26.7
HR (%/max)	85.5 ± 2.3	4.0 ± 3.0	91.3 ± 2.5	107 ± 2	**0** **.** **76**	1.8 ± 0.6	92.2 ± 2.6	108 ± 2	**0** **.** **71**	1.7 ± 0.6	93.7 ± 2.7	110 ± 3	**0** **.** **59**	1.5 ± 0.5
RPE (1–10)	7.9 ± 0.5	12.2 ± 4.2												

Correlation coefficients of match average and MDPs and CVs between matches.

* = statistically significant difference (*p* < 0.05) between the senior team and U18 team players. Bolded *r* values = statistically significant (*p* < 0.05) correlation coefficient. Bolded *g* values = large (>0.8) effect size between groups.

TD, total distance; HSRD, high-speed running distance; VHSRD, very-high-speed running distance; Pmet, metabolic power; HMLD, high-metabolic load distance; ACCD, acceleration distance; DECD, deceleration distance; HR, average heart rate; RPE, rate of perceived exertion.

**Table 2:** Average and MDPs in LSG demands of the senior team and U18 team players.

Senior team	LSG average				LSG 5-min MDP				LSG 1-min MDP			
	Mean + SD (*g*)	% of match average (*g*)	*r*	CV	Mean + SD (*g*)	% of match 5-min MDP (*g*)	*r*	CV	Mean + SD (*g*)	% of match 1-min MDP (*g*)	*r*	CV
TD (m/min)	127.5 ± 7.6# **(0.92)**	119 ± 6 (−0.04)	**0** **.** **69**	3.8 ± 2.4	137.1 ± 7.4 (0.41)	102 ± 5 (−0.24)	**0** **.** **76**	3.6 ± 2.2	166.9 ± 9.4# (0.27)	94 ± 6 (−0.19)	**0** **.** **55**	3.9 ± 2.5
HSRD (13–19 km/h) (m/min)	29.0 ± 6.0*# **(0.99)**	140 ± 22 (0.06)	**0** **.** **68**	13.9 ± 11.5	35.3 ± 6.6# (0.48)	91 ± 12 (−0.21)	**0** **.** **69**	16.7 ± 7.3	66.2 ± 13.2# (0.15)	83 ± 11 (−0.71)	**0** **.** **78**	9.5 ± 7
VHSRD (>19 km/h) (m/min)	4.7 ± 2.3* **(0.85)**	117 ± 49 (0.65)	**0.80**	24.9 ± 16.2	8.3 ± 3.9*# (0.65)	60 ± 24 (0.24)	**0** **.** **71**	29.8 ± 14.4	25.1 ± 8.0*# **(0.85)**	60 ± 16 (0.29)	**0** **.** **70**	33.7 ± 22.4
Pmet (W/kg)	8.4 ± 0.6*# **(1.01)**	122 ± 7 (−0.07)	**0** **.** **69**	4.0 ± 2.4	9.7 ± 0.6# (0.57)	104 ± 6 (0.04)	**0** **.** **68**	4.2 ± 2.6	12.0 ± 0.9 (0.46)	97 ± 7 (−0.07)	0.50	4.0 ± 3.1
HMLD (>20 W/kg) (m/min)	18.2 ± 3.8*# **(1.13)**	148 ± 22 (0.43)	**0** **.** **79**	12.4 ± 6.7	22.4 ± 4.5* (0.77)	96 ± 15 (0.13)	**0** **.** **67**	9.9 ± 6.3	44.3 ± 6.6# (0.67)	89 ± 11 (−0.09)	**0** **.** **62**	10.5 ± 6.9
ACCD (>2 m/s^2^) (m/min)	3.6 ± 1.0*# **(1.96)**	174 ± 32* **(1.26)**	**0** **.** **77**	20.5 ± 18.7	4.9 ± 1.5* **(1.52)**	110 ± 27 (0.65)	0.53	22.1 ± 19	10.8 ± 2.7* **(1.43)**	110 ± 24 (0.52)	0.45	20.5 ± 14.2
DECD (<−2 m/s^2^) (m/min)	3.4 ± 0.8*# **(1.36)**	178 ± 43* (0.63)	**0** **.** **69**	15.0 ± 12.6	4.6 ± 1.2* **(1.29)**	115 ± 28 (0.06)	**0** **.** **58**	16.3 ± 17.1	9.5 ± 2.2* **(1.09)**	107 ± 23 (−0.15)	**0** **.** **59**	16.5 ± 20.4
HR (%/max)	85.0 ± 2.2 (−0.06)	99 ± 3 (0.16)	**0** **.** **55**	2.8 ± 2.9	90.0 ± 2.2 (0.06)	99 ± 2 (0.45)	**0.80**	2.5 ± 2.8	92.5 ± 2.8 (−0.13)	99 ± 2 (−0.05)	**0** **.** **84**	2.0 ± 1.9
RPE (1–10)	5.5 ± 0.8# (0.10)	70 ± 10 (0.09)	0.35	15.2 ± 2.3								
Lactate (mmol/L)	5.5 ± 0.8 (−0.51)			32.1 ± 2.4								
U18 team	LSG average				LSG 5-min MDP				LSG 1-min MDP			
	Mean + SD	% of match average	*r*	CV	Mean + SD	% of match 5-min MDP	*r*	CV	Mean + SD	% of match 1-min MDP	*r*	CV
TD (m/min)	117.2 ± 13.6#	119 ± 9	**0** **.** **77**	4.0 ± 2.4	132.1 ± 15	104 ± 9	**0** **.** **71**	4.7 ± 2.3	162.9 ± 18.7	96 ± 8	**0** **.** **77**	4.7 ± 4.3
HSRD (13–19 km/h) (m/min)	22.7 ± 6.7#	138 ± 37	**0** **.** **56**	14.3 ± 10.6	31.3 ± 9.8	95 ± 20	**0** **.** **71**	14.4 ± 10.8	63.9 ± 17.2	96 ± 22	**0** **.** **64**	18.0 ± 11.1
VHSRD (>19 km/h) (m/min)	2.9 ± 2.2	85 ± 49	**0** **.** **79**	36.1 ± 27.5	5.7 ± 4.2#	52 ± 36	**0** **.** **62**	37.1 ± 20.3	17.3 ± 10#	52 ± 34	0.43	35.6 ± 22.8
Pmet (W/kg)	8.1 ± 0.9#	122 ± 11	**0** **.** **73**	4.0 ± 2.4	9.2 ± 1.0	104 ± 9	**0** **.** **69**	4.1 ± 3.4	11.5 ± 1.2	97 ± 8	**0** **.** **78**	3.5 ± 3.6
HMLD (>20 W/kg) (m/min)	13.6 ± 4.3#	135 ± 36	**0** **.** **63**	12.6 ± 9.3	18.3 ± 5.9	93 ± 22	**0** **.** **76**	12.4 ± 13.9	37.6 ± 12.0	91 ± 30	0.47	17.2 ± 14.3
ACCD (>2 m/s^2^) (m/min)	2.2 ± 0.5#	133 ± 32	**0** **.** **71**	14.1 ± 12.8	3.2 ± 0.7	93 ± 25	**0** **.** **52**	14.2 ± 11.4	6.0 ± 2.1	97 ± 25	0.28	31.2 ± 16.4
DECD (<−2 m/s^2^) (m/min)	2.3 ± 0.8#	151 ± 47	**0** **.** **71**	18.9 ± 16.5	3.2 ± 0.8	117 ± 59	**0** **.** **59**	10.7 ± 6.2	7.3 ± 1.9	119 ± 79	0.28	20.2 ± 11.6
HR (%/max)	85.1 ± 2.9	99 ± 3	0.48	3.4 ± 1.9	89.8 ± 2.9	98 ± 4	**0** **.** **78**	1.9 ± 1.0	92.9 ± 2.9	99 ± 3	**0** **.** **64**	1.6 ± 0.9
RPE (1–10)	5.4 ± 1.1#	69 ± 13	0.30	14.1 ± 6.1								
Lactate (mmol/L)	6.2 ± 1.7			27.6 ± 5.1								

Correlation coefficients of variables between LSG and CVs between test sessions.

Statistically significant difference (*p* < 0.05): * = between the senior team and U18 team players. Bolded *g* values = large (>0.8) effect size between groups. Statistically significant difference (*p* < 0.05): # = between LSG and match play. Bolded *r* values = statistically significant (*p* < 0.05) correlation coefficient between LSG and match play.

**Table 3:** Average and MDPs in SSG demands of the senior team and U18 team players.

Senior team	SSG average				SSG 3-min MDP				SSG 1-min MDP			
	Mean + SD (*g*)	% of match average (*g*)	*r*	CV	Mean + SD (*g*)	% of match 3-min MDP (*g*)	*r*	CV	Mean + SD (*g*)	% of match 1-min MDP (*g*)	*r*	CV
TD (m/min)	101.0 ± 7.2# (0.59)	94 ± 6 (−0.62)	**0** **.** **55**	5.6 ± 7.9	120.2 ± 10.5*# **(0.89)**	83 ± 5 (−0.07)	**0** **.** **75**	4.1 ± 3.1	139.1 ± 11.2*# **(0.97)**	78 ± 6 (0.15)	**0** **.** **53**	4.0 ± 3.1
HSRD (13–19 km/h) (m/min)	14.6 ± 5.4*# **(1.35)**	69 ± 21 (0.58)	**0** **.** **60**	5.8 ± 6.7	23.8 ± 7.6*# **(0.99)**	50 ± 15 (0.29)	0.40	6.9 ± 4.1	40.2 ± 11.2*# **(1.32)**	49 ± 11 (0.50)	**0** **.** **54**	9.7 ± 6.6
VHSRD (>19 km/h) (m/min)	0.3 ± 0.3# (−0.17)	14 ± 11 (−0.49)	0.23	135.5 ± 16.8	1.1 ± 1.2# (−0.24)	11 ± 7 (−0.27)	−0.11	130.9 ± 29.7	3.1 ± 3.5# (−0.17)	14 ± 10 (−0.16)	−0.19	126.1 ± 43.4
Pmet (W/kg)	7.6 ± 0.5* **(0.84)**	103 ± 6 (−0.54)	**0** **.** **63**	6.0 ± 7.9	9.2 ± 0.8*# **(1.05)**	91 ± 6 (0.35)	**0** **.** **72**	3.8 ± 2.2	10.7 ± 0.8*# **(1.02)**	85 ± 7 (0.33)	0.49	4.0 ± 3.2
HMLD (>20 W/kg) (m/min)	12.9 ± 3.8* **(1.30)**	102 ± 19 (0.46)	**0** **.** 83*	11.3 ± 8.7	19.5 ± 5.3*# **(1.36)**	65 ± 15 (0.53)	**0** **.** 64*	11.4 ± 9.8	30.1 ± 8.9*# **(1.18)**	59 ± 13 (0.32)	**0** **.** 64*	12.7 ± 12.4
ACCD (>2 m/s^2^) (m/min)	4.6 ± 1.3*# **(0.90)**	215 ± 45 (−0.27)	**0** **.** 75*	12.8 ± 13.9	7.6 ± 1.7*# **(1.29)**	134 ± 22 (0.03)	**0** **.** 71*	11.9 ± 10.2	12.3 ± 2.9*# **(1.19)**	119 ± 20 (−0.05)	**0** **.** 75*	8.6 ± 9.2
DECD (<−2 m/s^2^) (m/min)	3.4 ± 0.9*# **(1.17)**	174 ± 35 (0.16)	**0** **.** **72**	8.0 ± 7.9	5.3 ± 1.4* **(1.27)**	106 ± 25 (0.14)	0.47	10.4 ± 7.5	8.7 ± 1.7* **(1.42)**	98 ± 19 (−0.04)	0.45	8.6 ± 5.6
HR (%/max)	85.6 ± 2.4 (0.24)	100 ± 3 (0.21)	**0** **.** **52**	2.2 ± 1.3	92.0 ± 2.1 (0.55)	101 ± 2 (0.53)	0.43	2.6 ± 2.3	93.6 ± 1.7 (0.36)	100 ± 3 (0.40)	−0.02	2.7 ± 2.3
RPE (1–10)	6.7 ± 1.0# (0.50)	86.1 ± 12.9 (0.97)	0.47	12.2 ± 1.3								
Lactate (mmol/L)	8.0 ± 2.1 (0.44)			14.4 ± 1.6								
U18 team	SSG average				SSG 3-min MDP				SSG 1-min MDP			
	Mean ± SD	% of match average	*r*	CV	Mean ± SD	% of match 3-min MDP	*r*	CV	Mean ± SD	% of match 1-min MDP	*r*	CV
TD (m/min)	96.5 ± 7.7	98 ± 7	**0** **.** **65**	10.2 ± 5.1	111.9 ± 8.3#	83 ± 6	**0** **.** **55**	5.2 ± 5.8	128.6 ± 10.7#	77 ± 6	0.47	10.2 ± 15.6
HSRD (13–19 km/h) (m/min)	8.5 ± 3.7#	55 ± 26	0.21	26.4 ± 18	16.0 ± 8.2#	44 ± 29	−0.05	18.8 ± 14.9	26.7 ± 9.2#	42 ± 16	0.26	35.7 ± 37.0
VHSRD (>19 km/h) (m/min)	0.4 ± 0.4#	20 ± 22	0.02	109.3 ± 37.3	1.4 ± 1.3#	13 ± 9	0.18	100.5 ± 50.4	3.7 ± 3.8#	16 ± 12	0.12	94.6 ± 50.4
Pmet (W/kg)	7.0 ± 0.9#	107 ± 10	**0** **.** **59**	10.8 ± 5.8	8.4 ± 0.8#	89 ± 6	**0** **.** **72**	7.6 ± 11.0	9.8 ± 0.9#	83 ± 6	**0** **.** **61**	9.0 ± 10.0
HMLD (>20 W/kg) (m/min)	8.7 ± 2.7	91 ± 30	0.42	21.4 ± 13.8	12.9 ± 4.5#	55 ± 22	0.15	26.2 ± 36.4	21.4 ± 6.8#	53 ± 20	0.17	26.8 ± 38.9
ACCD (>2 m/s^2^) (m/min)	3.6 ± 1.0#	235 ± 92	0.26	23.6 ± 14.9	5.6 ± 1.4	133 ± 51	0.12	19.8 ± 14.9	9.3 ± 2.1	120 ± 40	0.09	17.1 ± 18.5
DECD (<−2 m/s^2^) (m/min)	2.3 ± 0.9#	165 ± 69	0.38	19.3 ± 14.5	3.6 ± 1.3	101 ± 49	0.23	18.6 ± 19.7	6.0 ± 2.1	99 ± 54	0.02	18.8 ± 18.4
HR (%/max)	85.1 ± 2.2	99 ± 3	0.28	5.6 ± 4.7	90.4 ± 3.5	99 ± 3	0.32	1.7 ± 1.7	92.7 ± 3.1	99 ± 3	0.35	1.5 ± 1.2
RPE (1–10)	6.2 ± 1.0	68.5 ± 21.7	0.19	16.6 ± 4.7								
Lactate (mmol/L)	7.3 ± 2.3			28.3 ± 3.8								

Correlation coefficients of variables between SSGs and matches and CVs between n test sessions.

Statistically significant difference (*p* < 0.05): * = between the senior team and U18 team players. Bolded *g* values = large (>0.8) effect size between groups. Statistically significant difference (*p* < 0.05): # = between LSG and match play. Bolded *r* values = statistically significant (*p* < 0.05) correlation coefficient between SSG and match play.

Pearson's product–moment correlation was used to assess the correlations between match average and 1-, 3-, and 5-min MDPs and to assess the correlations between match average and SSG and LSG average and match MDPs and SSG and LSG MDPs. The correlation magnitudes were classified using the following criteria: <0.3, weak; 0.3–0.7, moderate; and >0.7, strong. A linear regression model (*y* = *x* + *g* + *xg*) was used to evaluate the possible differences between the magnitudes of correlation coefficients of each group, where *y* is the senior team correlate, *x* is the U18 team correlate, *g* is the group, and *xg* is the interaction.

## 3. Results

### 3.1. Match demands

Match demands increased significantly in all variables of both groups: average <5-min MPD <3-min MDP <1-min MDP. Table 1 shows that the senior team players reached significantly higher average and MDP values in many of the variables compared with those of the U18 team players. However, in MDP values, relative to match average values, there were significant between-group differences observed in only 5-min and 1-min total distance (TD) and 5-, 3-, and 1-min Pmet MDPs relative to average match values (Figure S1 in Supplementary Material).

The correlation coefficients of both groups between match average and selected time window MDP values were significant and strong in all variables except in 1-min VHSRD and HR where they were moderate. Coefficients of variation (CVs) varied depending on the variable in question: range = 1.5% in 1-min MDP HR of the U18 team and 42.4% in 3-min MDP VHSRD of the U18 team.

### 3.2. LSG and SSG demands

LSG demands increased significantly in all variables of both groups: average <5-min MPD <3-min MDP <1-min MDP. Table 2 shows that in LSGs, the senior team players reached significantly higher values in average, 5-, and 1-min MDPs of VHSRD, ACCD, and DECD compared with those of the U18 team players. This between-group difference was also observed in average HSRD, Pmet, and 5-min MDP of HMLD. In these MDP values relative to the match MDP values of the players, the only between-group differences that remained were in the ACCD and DECD averages. LSG average TD, ACCD, DECD, HSRD, Pmet, and HMLD were higher in both groups compared with those during the matches. Conversely, in both groups, LSG 5- and 1-min VHSRD MDPs, as well as TD 1-min MDP of the senior team were lower compared with those during the matches (Figure S2 in Supplementary Material). The correlation coefficients of both groups between selected time window LSG and match values varied from moderate to strong. CVs varied between 1.6% (HR 1-min MDP of the U18 team) and 37.1% (VHSRD 5-min MDP of the U18 team).

SSG demands increased significantly in all variables of both groups: average <5-min MPD < 3-min MDP < 1-min MDP. Table 3 shows that in SSGs, the senior team players reached significantly higher values in ACCD, DECD, HSRD, Pmet, and HMLD averages and 3- and 1-min MDPs in TD, ACCD, DECD, HSRD, Pmet, and HMLD compared with those of the U18 team players. No differences between groups in these values relative to match values were found. SSG average ACCD and DECD in both groups, 3- and 1-min ACCD MDPs of the senior team, and average Pmet of the U18 team were higher compared with those during the matches. Instead, SSG HSRD and VHSRD in all time windows; 3- and 1-min TD, HMLD, and Pmet MDPs in both groups; and the average TD of the senior team were lower compared with those during the matches. Correlation coefficients between selected time windows in SSGs and matches varied from weak to strong. The senior team players demonstrated significantly higher correlation coefficients in average, 3-, and 1-min MDPs of ACCD and HMLD compared with those of the U18 team players, as assessed by linear regression modeling. CVs varied between 1.5% (HR 1-min MDP of the U18 team) and 135.5% (average VHSRD of the senior team).

## 4. Discussion

The aim of the study was (1) to determine the average and MDPs of national-level female soccer matches and (2) to evaluate the relationship between average and MDPs during SSGs, LSGs, and matches. The findings of the present study showed that the average demands of the official matches heavily underestimate the MDPs of national-level female soccer players. However, the correlation coefficients between match average and MDP values were mainly strong, indicating that traditionally used match averages and recently proposed MDP values reflect similar behaviors in female soccer players. LSGs seem to offer a training stimulus that overloads match average demands in TD, HSRD, Pmet, HMLD, ACCD, and DECD. For the senior team players, LSGs underload match MDPs of TD, HSRD, VHSRD, and HMLD, whereas LSGs replicate match MDPs of the U18 team players in most of the variables assessed. SSGs, instead, can be used to overload or replicate match average and MDP demands of ACCD and DECD in both groups, but ACCD and HMLD of the senior team were more associated with the match demands than those of the U18 team. Thus, SSGs may be a more appropriate training tool for the senior team players.

Overall, match average and MDP demands from the present study were similar to those previously reported in national-level female soccer matches (4, 8, 25). As expected, the senior team players reached significantly higher values in almost all match external load variables, both average (3, 26) and MDPs, compared with those of the U18 team players. Interestingly, when MDPs were relative to the match average values of each player, the only significant differences between groups were the higher TD and Pmet of the U18 team players. Minor differences between groups in relative MDP values are logical because significant and strong correlations (*r* = 0.71–0.97) were found in external load variables between match average and MDPs. Thus, the average performance of the players during the match was highly associated with their performance during MDPs.

External load variables during 1-min, 3-min, and 5-min MDPs were 167%–1,165%, 135%–504%, and 126%–359%, of match average values, respectively. The previous studies conducted on elite male and female players have shown that, due to the intermittent nature of soccer match play, the whole match average demands heavily underestimate the highest intensity that players perform in the match (7, 24). In addition, the present study replicated those findings now in national-level female players. Ultimately, the coaches and practitioners should be aware of the level of match MDPs to be able to prescribe training that prepares players for the most intensive periods of the match.

LSGs overloaded all external load variables, except for VHSRD, compared with those during the matches when average values were analyzed. Senior teams’ 5- and 1-min HSRD, VHSRD, and HMLD MDPs were underloaded compared with those during the matches, while only VHSRD was underloaded in the U18 team. This indicates that the senior team players likely need more specific training than LSGs to reach match MDPs of HSRD, HMLD, and VHSRD. Thus, from a tactical periodization perspective, LSGs can be recommended for national-level female players to overload only average match demands of average running volume (TD) and mechanical load (ACCD and DECD). Higher training intensity in LSGs or more specific games or drills would be required to overload MDPs relative to matches. Similarly, LSGs can be recommended to replicate average match VHSRD, but a different training method would be needed to overload average VHSRD or replicate MDP VHSRD. In general, the findings in the senior team were similar to those reported in elite male players, where TD and HSRD in 8 vs. 8 LSGs are underloaded in 1-min to 15-min MDPs compared with matches, while acceleration and deceleration demands replicate match demands (14).

The average ACCD and DECD of both groups and average Pmet of the U18 team were overloaded in SSGs compared with those during the match averages, while average HSRD and VHSRD and the average TD of the senior team were underloaded. When considering MDPs, SSGs replicated and overloaded match ACCD of the U18 team and senior teams, respectively, and replicated the match DECD MDPs of both groups. Other MDP external load variables were underloaded in SSGs compared with those during the matches. Thus, SSGs could be recommended to overload or replicate match average and MDPs of ACCD and DECD, but larger games or specific drills must be needed to overload or replicate other external load variables. In the context of tactical periodization, these findings suggest that SSGs can be recommended for national-level female players where desired. Similar findings have been reported in elite male players where TD and HSRD during MDPs (<4-min) of 4 vs. 4 SSGs were lower compared with those in the matches, and acceleration and deceleration demands were higher than those in the matches (14).

The correlation coefficients between the external load variables of match average, 3-min, and 1-min and the same time windows from SSGs varied from weak to strong (*r *= 0.02–0.83) and LSGs from moderate to strong (*r *= 0.43–0.84). In previous studies conducted on male youth players, higher (15) and lower (16) associations in external load variables between SSGs and matches have been reported compared with those found in the present study. The senior team players reached significantly higher correlation coefficients between match and SSG average and MDP demands in HMLD and ACCD than those of the U18 team players; hence, SSGs offered a more match-specific stimulus to the senior team players in acceleration distance and distance covered in high metabolic load compared with those of the U18 team players. In other variables, SSG and LSG offered similar stimuli relative to match load for both senior and U18 team players, even though the senior team players reached significantly higher values in several variables during SSGs and LSGs.

In matches, the only significant difference between groups in internal load (HR, RPE, lactate) was −0.2 arbitrary units of higher RPE of the senior team after matches. In SSGs and LSGs, there were no differences between groups in internal load variables, suggesting that SSGs and LSGs offered similar cardiorespiratory and perceived training stimuli to the senior team and U18 team players. Simultaneously, the senior team players reached higher values in almost all matches and several SSG and LSG external load variables compared with the U18 team players. Thus, the senior team players obtained higher work at a similar physiological cost compared with U18 team players during all formats. One logical explanation for these findings could be that the senior team players had better physical qualities, which would have allowed them to reach higher external load for a similar internal load. However, as running performance during soccer matches is a complex phenomenon, other contextual aspects might have played a role leading to this difference (6).

Variability of match, SSG, and LSG data was estimated by calculating CVs, and, in general, findings were in line with the findings of the previous literature (8, 27). Similar to the findings of external load variables between matches in elite players, the variability was lowest in total distance and highest in VHSRD for both average and 5-min MDP values (27). CVs were smaller in match average values than match MDP values, suggesting that match-to-match average demands vary less than match-to-match MDPs. Compared with previous findings from elite female players, 1-, 3-, and 5-min MDP CVs were slightly lower in the present study (8), indicating less between-match variation in the national-level matches of the studied teams. In LSGs and SSGs, CVs were similar to matches, except for the extremely high CV in VHSRD (95%–136%) during SSGs. Such a result is due to the amount of VHSRD in SSGs being low, e.g., one additional short sprint during a SSG can cause high CVs, and even higher CVs have been reported from SSGs played by male players (28).

One strength of the present study was a novel approach in female soccer research comparing both average and MDP demands of the match with such demands during SSGs and LSGs. The previous studies have mostly focused on external load variables. Thus, the measurement of internal load variables offered insight into the psychophysical response of female soccer players to external load during matches and various-sided games, which are important for potential training adaptations. Finally, analysis of match, SSG, and LSG CVs showed the day-to-day variation when playing/training in soccer, and, encouragingly for training prescription, variations of SSGs and LSGs were generally slightly lower compared with those during matches.

The biggest limitation of this study was that the playing positions were not able to be taken into account in the analyses due to the relatively low sample size. The second major limitation was that only two teams from a single club participated in the study; thus, the findings are representative of, e.g., the philosophy, playing style, and tactics of this club. More research is needed to generalize the results from a wider domestic female soccer population and to investigate the potential effects of contextual factors, such as playing position or team formation.

The present study showed that SSGs and LSGs can overload selected variables relative to match average load; however, examining whether systematic overload leads to greater development than lower load a randomized controlled trial is needed in the future.

## 5. Conclusions

MDPs during national-level female soccer matches are higher compared with average demands, which should be taken into account in training prescription. SSGs can offer a training stimulus that overloads average match demands in acceleration and deceleration distances and overloads or replicates match MDPs in these variables. Thus, in the context of tactical periodization, SSGs can be used in training to target acceleration/deceleration. Alternatively, LSGs can be used to overload average match demands in total, high-speed running, acceleration, deceleration, and high-metabolic load distances, as well as average metabolic power. LSGs can also be used to replicate match MDPs of the U18 team players in these variables. LSGs in isolation may be suboptimal for the senior team players in relation to match MDPs of high-speed running, very-high-speed running, and high-metabolic load distances; thus, a different training strategy could be explored in the future.

## Data Availability

The raw data supporting the conclusions of this article will be made available by the authors, without undue reservation.

## Supplementary material

Supplementary Figure S1 On left panel 1^st^-team and U18-team absolute match average and most demanding passage (MDP) values in total distance (TD), very-high-speed running distance (VHSRD), acceleration distance (ACCD) and deceleration distance (DECD). On right panel same variables' MDPs relative to player's match average. Dashed line represents match values. * = Statistically significant difference (p < 0.05) between 1st- and U18-team players.

Supplementary Figure S2 On left panel 1^st^-team and U18-team player's absolute small-(SSG) and large-sided-games (LSG) average and most demanding passages (MDP) values in total distance (TD), very-high-speed running distance (VHSRD), acceleration distance (ACCD) and deceleration distance (DECD). On right panel same variables' MDPs relative to player's match values. Dashed line represents match values. * = Statistically significant difference (p < 0.05) between 1st-team and U18-team players.
