# Supplementary figures and images for: Average demands and most demanding passages of national-level female soccer matches: do small- and large-sided games replicate match demands?
Source: Front Sports Act Living. 2023 Oct 11;5:1236112. doi: 10.3389/fspor.2023.1236112 (PMC10598713; doi:10.3389/fspor.2023.1236112)

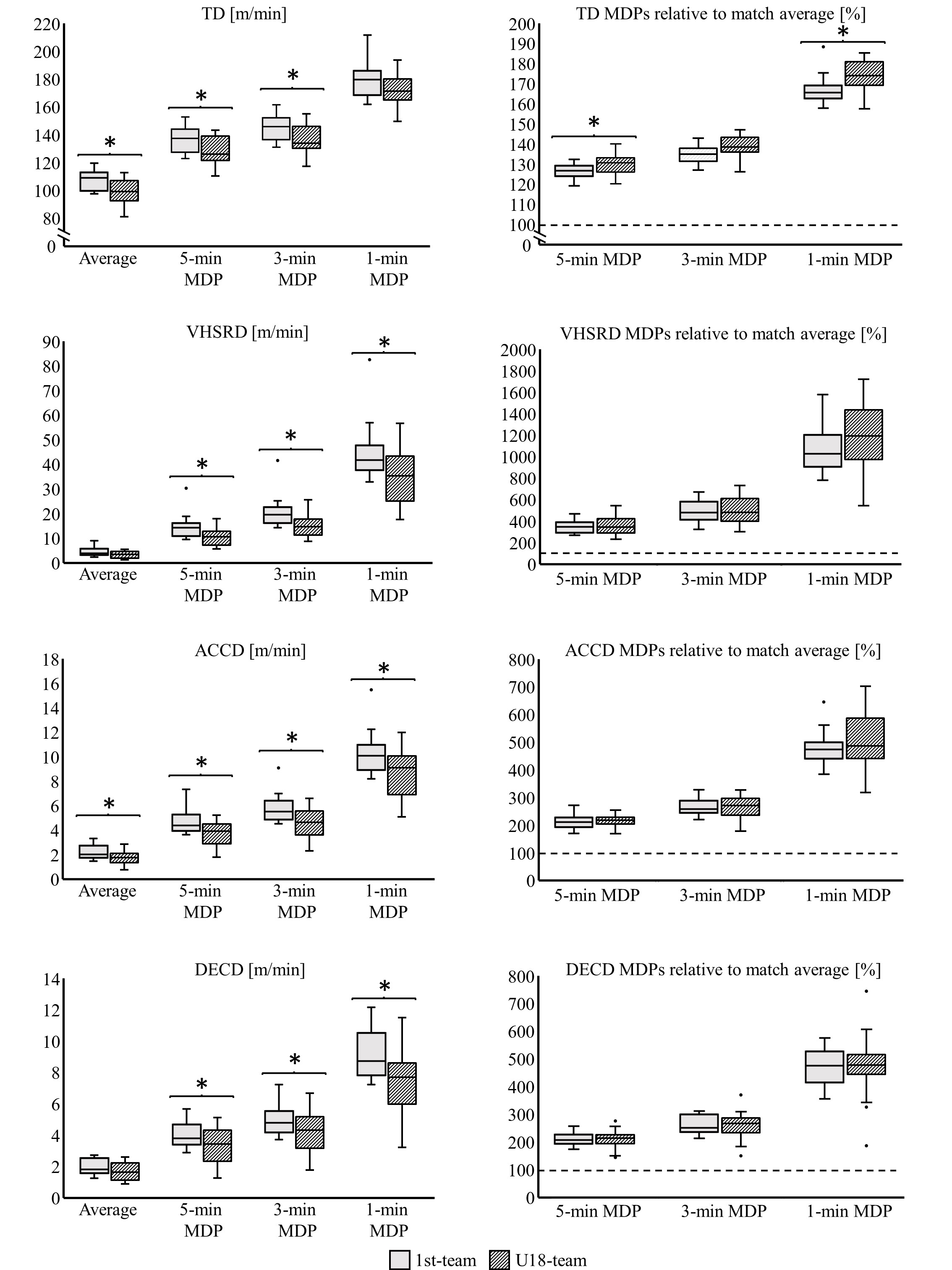

Supplement: Supplementary Figure S1 — On left panel 1st-team and U18-team absolute match average and most demanding passage (MDP) values in total distance (TD), very-high-speed running distance (VHSRD), acceleration distance (ACCD) and deceleration distance (DECD). On right panel same variables' MDPs relative to player's match average. Dashed line represents match values. * = Statistically significant difference (p < 0.05) between 1st- and U18-team players. [file Image1.jpeg]

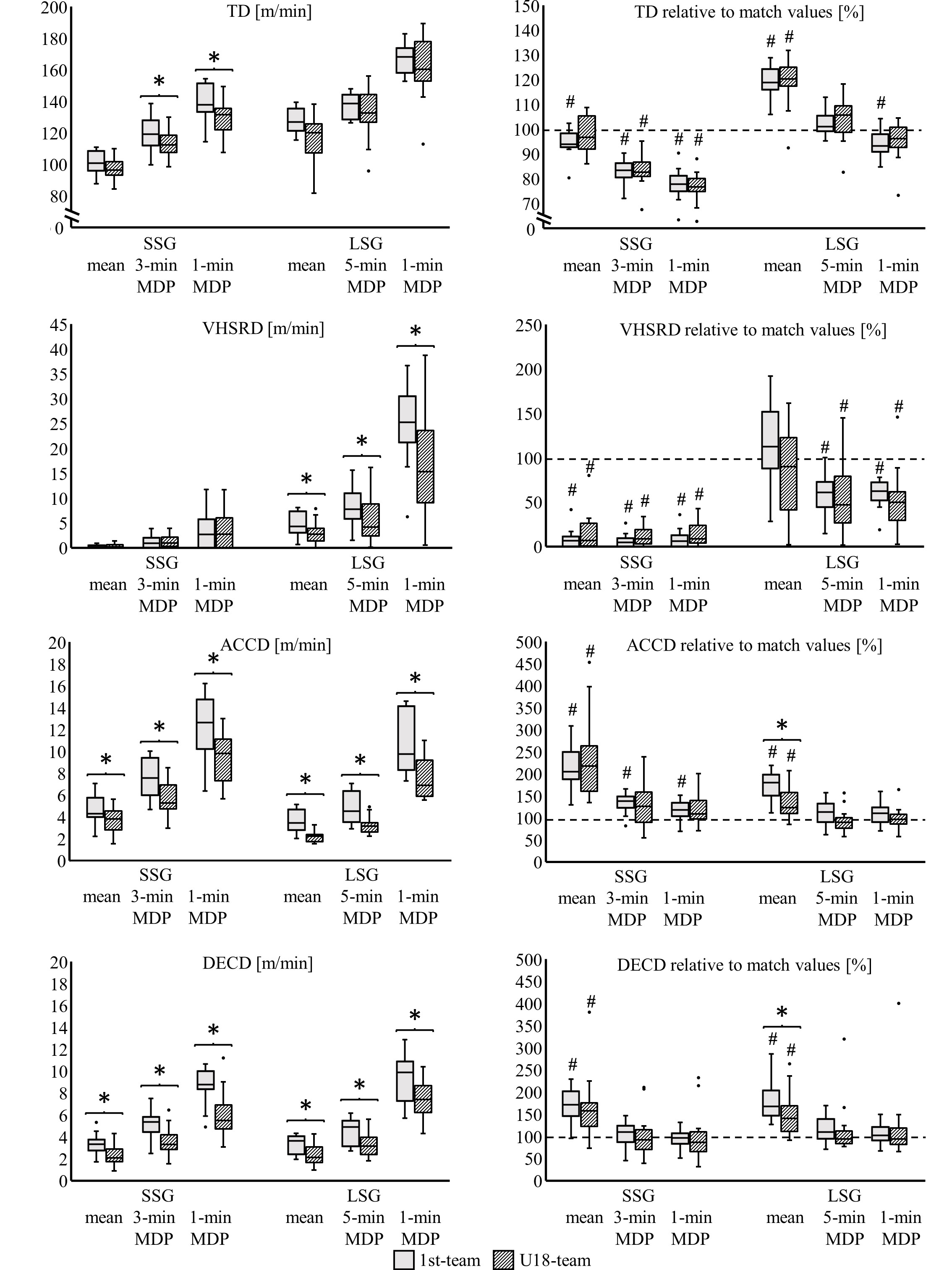

Supplement: Supplementary Figure S2 — On left panel 1st-team and U18-team player's absolute small-(SSG) and large-sided-games (LSG) average and most demanding passages (MDP) values in total distance (TD), very-high-speed running distance (VHSRD), acceleration distance (ACCD) and deceleration distance (DECD). On right panel same variables' MDPs relative to player's match values. Dashed line represents match values. * = Statistically significant difference (p < 0.05) between 1st-team and U18-team players. [file Image2.jpeg]
